# Draft genome of *Dugesia japonica* provides insights into conserved regulatory elements of the brain restriction gene *nou-darake* in planarians

**DOI:** 10.1186/s40851-018-0102-2

**Published:** 2018-08-29

**Authors:** Yang An, Akane Kawaguchi, Chen Zhao, Atsushi Toyoda, Ali Sharifi-Zarchi, Seyed Ahmad Mousavi, Reza Bagherzadeh, Takeshi Inoue, Hajime Ogino, Asao Fujiyama, Hamidreza Chitsaz, Hossein Baharvand, Kiyokazu Agata

**Affiliations:** 1 Department of Biophysics, Kyoto University, Kyoto, Japan; 2 Department of Animal Bioscience, Nagahama Institute of Bio-Science and Technology, Nagahama, Japan; 3 School of Pharmacy, Fudan University, Shanghai, China; 4 Comparative Genomics Laboratory, National Institute of Genetics, Mishima, Japan; 5 Department of Computer Science, Colorado State University, Fort Collins, USA; 6 Department of Stem Cells and Developmental Biology, Cell Science Research Center, Royan Institute for Stem Cell Biology and Technology, ACECR, Tehran, Iran; 7 Department of Computer Engineering, Sharif University of Technology, Tehran, Iran; 8 Department of Developmental Biology, University of Science and Culture, Tehran, Iran; 9 Present address: Immolife-biotech Co., Ltd., Nanjing, China; 10 Present address: Research Institute of Molecular Pathology (IMP), Vienna, Austria; 11 Present address: Department of Life Science, Gakushuin University, Tokyo, Japan; 12 Present address: Amphibian Research Center, Hiroshima University, Higashi-hiroshima, Japan; 13 Institute of Neurogenomics, Helmholtz Zentrum München, German Research Centre for Environmental Health, Neuherberg, Germany

**Keywords:** Planarian, *Dugesia japonica*, Genome, Conserved non-coding elements, *Nou-darake*

## Abstract

**Background:**

Planarians are non-parasitic Platyhelminthes (flatworms) famous for their regeneration ability and for having a well-organized brain. *Dugesia japonica* is a typical planarian species that is widely distributed in the East Asia. Extensive cellular and molecular experimental methods have been developed to identify the functions of thousands of genes in this species, making this planarian a good experimental model for regeneration biology and neurobiology. However, no genome-level information is available for *D. japonica*, and few gene regulatory networks have been identified thus far.

**Results:**

To obtain whole-genome information on this species and to study its gene regulatory networks, we extracted genomic DNA from 200 planarians derived from a laboratory-bred asexual clonal strain, and sequenced 476 Gb of data by second-generation sequencing. Kmer frequency graphing and fosmid sequence analysis indicated a complex genome that would be difficult to assemble using second-generation sequencing short reads. To address this challenge, we developed a new assembly strategy and improved the de novo genome assembly, producing a 1.56 Gb genome sequence (DjGenome ver1.0, including 202,925 scaffolds and N50 length 27,741 bp) that covers 99.4% of all 19,543 genes in the assembled transcriptome, although the genome is fragmented as 80% of the genome consists of repeated sequences (genomic frequency ≥ 2). By genome comparison between two planarian genera, we identified conserved non-coding elements (CNEs), which are indicative of gene regulatory elements. Transgenic experiments using *Xenopus laevis* indicated that one of the CNEs in the *Djndk* gene may be a regulatory element, suggesting that the regulation of the *ndk* gene and the brain formation mechanism may be conserved between vertebrates and invertebrates.

**Conclusion:**

This draft genome and CNE analysis will contribute to resolving gene regulatory networks in planarians. The genome database is available at: http://www.planarian.jp.

**Electronic supplementary material:**

The online version of this article (10.1186/s40851-018-0102-2) contains supplementary material, which is available to authorized users.

## Background

Planarian is a common name applied to species of non-parasitic Platyhelminthes (flatworms) of the turbellaria class. Planarians have attracted great interest in the field of regeneration biology for many years [1]. *Dugesia japonica* (*D. japonica*) is a typical freshwater planarian species that is widely distributed in East Asia [2]. This planarian is able to regenerate a complete individual from a tiny excised part of its body, which makes it a good model for regeneration biology and regenerative medicine research [3]. *D. japonica* also has a well-organized brain and shows decision-making behavior [4–6], and is rapidly becoming a model animal used in the study of neurobiology [7–10].

Advances in cellular and molecular biology experimental methods, as well as nucleic acid sequencing technologies, have helped increase our ability to examine the biology of planarians at the molecular level. Using PCR, cDNA libraries, RNA in situ hybridization and immunoscreening, *D. japonica* cell-type-specific genes have been isolated [11]. Highly sensitive in situ hybridization methods were developed for identifying mRNA locations and expression in particular cell types [5]. Loss-of-function assays, including RNA interference, were also devised to characterize gene functions [12]. RNA microarrays have been generated to identify genes important for regeneration, and head-specific genes [13]. Fluorescence-activated cell sorting (FACS) has been used to isolate discrete cell populations, which could then be used for single-cell gene profiling, functional transplantation studies, and neurobiology studies [14–17]. In addition, a transcriptome resource (EST data) is also available [18, 19]. All of these modern research methods and resources have enabled us to link the phenomena of *D. japonica*’s robust regeneration and brain formation to underlying genes. To achieve a complete understanding of those genes and their regulatory networks, genome-level information on *D. japonica* is required. However, the unusual number of SNPs that accumulate during asexual reproduction has confounded our efforts to obtain long contigs, although we used the laboratory clonal strain of *D. japonica* [19]. Here we present the first draft genome from only second-generation sequencing data, made possible by overcoming heterogenesis by using a newly developed assembly strategy, and analysis of conserved non-coding elements (CNEs) of *D. japonica*. These results will contribute knowledge essential for further research into gene regulatory networks in planarian.

## Methods

### Animal culture and DNA extraction

The asexual *D. japonica* strain SSP-9 T-5 was derived from a single individual and maintained in the Agata lab since 2005 [19]. The planarians were kept in autoclaved tap water at 22–24 °C in dim light, fed with chicken liver twice a week, and starved for at least 1 week before experiments. Genomic DNA was extracted using the Wizard® SV Genomic DNA Purification System from Promega.

### Kmer analysis and genome size estimation

Kmers were counted in the Illumina Hiseq sequencing data using Jellyfish with the “–C” parameter [20]. Genome size was estimated by the formula G = N_base_/C_base_ = N_Kmer_/C_Kmer_ (G is genome size, N_base_ and N_Kmer_ are the numbers of bases and Kmers, and C_base_ and C_Kmer_ are expected sequencing depth of bases and Kmers).

### Genome assembly and annotation

The preliminary genome de novo assembly was performed by *de Brujin* graph-based method, Allpth-lg [21], SOAPdenovo [22], and Velvet [23]. To improve the assembly, a new strategy was used, containing four steps. At first, all pair-end sequencing reads were locally assembled into precise pseudo-long reads by AnyTag [24]. Secondly, all Sanger long reads, Roche 454 long reads, and newly produced pseudo-long reads were assembled into contigs using an overlap-layout algorithm, Newbler [25]. Furthermore, Illumina mate-pair sequencing reads were used to link the contigs and generate scaffolds by SSPASE [26]. Thirdly, all pair-end and mate-pair information from the sequencing was utilized for gap closure by GapFiller [27]. Finally, super-scaffolds were generated by L_RNA_Scaffolder [28] based on RNA evidence. Genome annotation was performed using MAKER [29].

### Transcriptome assembly and annotation

The independent transcriptome de novo assembly was done by Trinity [30]. The transcriptome annotation was performed using Trinotate [31].

### Discovery of CNEs

Only scaffolds with mRNA evidence were selected by BLAT [32]. Coding regions were masked out by the letter “N” from the selected scaffolds using bedtools [33]. Repeated sequences were subsequently masked out by the letter “N” from the previously selected scaffolds using RepeatMasker (http://www.repeatmasker.org/). One-to-one matched scaffold pairs between the two planarian genomes were identified using NUCmer [34]. For specific genes of interest, additional Blastn procedures (both local Blastn [35] and online Blastn at the website of *Schmidtea mediterranea* (*S. mediterranea*) genome database [36]) were performed to refine the results, and finally, CNEs on the scaffolds were located.

### Enhancer activity assay using Xenopus embryos

The reporter plasmid, actGFP, carrying a chicken *β-actin* basal promoter (− 55 to + 53), was previously described as βGFP [37]. Non-coding sequences conserved between *D. japonica* and *S. mediterranea ndk* genomic regions were cloned in the actGFP from *D. japonica* genomic DNA by polymerase chain reaction (PCR) and verified by sequencing. Searches for putative transcription-factor-binding motifs were performed with transcription-factor-binding sites collected from the TRANSFAC and JASPAR databases [38, 39]. Transgenic *Xenopus* embryos were generated with a sperm nuclear transplantation method, as described [40]. The fraction of embryos that developed normally until scoring stages (stages 13–14) was subjected to in situ hybridization for detecting GFP expression with maximum sensitivity.

## Results

### Genome sequencing

For genome sequencing, we used a pool of around 200 planarian individuals from an asexually propagating clonal strain of *D. japonica* [19]. After extracting genomic DNA, we prepared short insert (~ 180 bp, 200 bp, 250 bp, and 450 bp) pair-end DNA libraries and large insert (~ 3 kb, ~ 8 kb, ~ 20 kb) mate-pair libraries. Three second-generation sequencing (SGS) platforms (Illumina GAIIx, Illumina Hiseq2000 and Roche 454 sequencing) were used. We also constructed a genomic DNA fosmid library of *D. japonica* with insert length ~ 35 kb*,* and 172 fosmids were sequenced by end-sequencing using a first-generation (Sanger) sequencing platform. Totally, 476.48 Gb data were generated (Table 1).

**Table 1:** Summary of library construction and sequencing

Resource	Insert Length (bp)	Average Read Length (bp)	Total Raw Data (Gb)
Illumina GAIIx	180	150 × 2	37.98
	200	150 × 2	26.54
	250	150 × 2	24.77
Illumina Hiseq2000	250	100 × 2	125.53
	450	100 × 2	58.81
	450	34 × 2	43.51
	3000	34 × 2	69.78
	8000	34 × 2	46.22
	20,000	34 × 2	36.91
Roche 454	3000	350 × 2	0.79
	8000	270 × 2	0.64
	Shotgun	400	2.62
	Shotgun	600	2.31
Sanger	35,000	1000 × 2	0.07
Total			476.48

### Kmer frequency analysis and genome size estimation

**Fig. 1:**
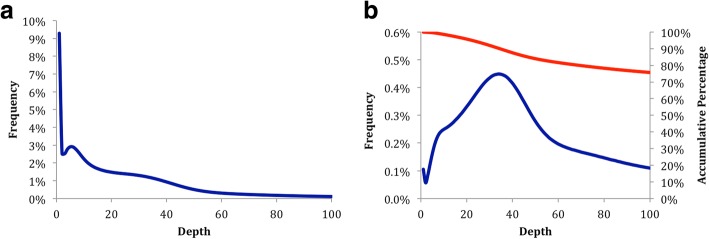
Kmer (k = 17) frequency analysis. **a** Kmer species frequency graph. The horizontal axis shows the depth of kmer species, and the vertical axis shows the percentage of each kmer species value (blue curve). **b**. Kmer individuals frequency graph. The horizontal axis shows depth of kmer individuals, the left vertical axis shows the percentage of each kmer individual’s value (blue curve), and the right vertical axis shows the accumulative frequency of the kmer individuals (red curve)

After quality control of the raw sequencing data, the average Q value of bases of sequencing reads was greater than 30 (Additional file 1), which indicated clean data with low sequencing error. Probably, due to the lack of sequencing data and the sampling algorithm of Kmergenie [41], previous kmer frequency analysis using only Illumina GAIIx data did not give a clean kmer species distribution peak [19]. After using more Hiseq 2000 pair-end sequencing data (from insert library 250 bp (2nd) and insert library 450 bp, Table 1), we calculated the kmer (k = 17) species and kmer individuals frequency graphs (Fig. 1) using the Jellyfish software [20], and generated 51.6G correct kmers in total. Although relatively clear peaks were shown, these graphs appeared unusual. The kmer species and individual graphs should be very similar after analyzing sequencing data from an ideal genome that has little heterozygosity and few repeated sequences [42], but these two graphs derived from *D. japonica* genome sequencing data were clearly different. In the kmer species frequency graph, the conspicuous high peak and the large number of kmer species before the unapparent low homozygous peak indicated *D. japonica* genome indicates a highly heterogeneous genome (Fig. 1a). The kmer individuals frequency graph (Fig. 1b) has a main peak at 34 (genomic frequency = 1), and depth of more than 80% of the kmers are higher than 68 (genomic frequency ≥ 2), indicating that the *D. japonica* genome is a highly repetitious genome in which about 80% of the genome comprises repeated sequences [42]. Accordingly, *D. japonica* may have a highly complex genome structure.

The *D. japonica* genome is normally a diploid genome that contains eight pairs of chromosomes (2n = 16). According to these kmer frequency graphs, the estimated *D. japonica* genome size is ~ 1.52 Gb. The *D. japonica* genome commonly has 16 chromosomes, twice the number of another planarian species, *S. mediterranea* (2n = 8, with an estimated genome size 769.5 Mb from Spencer Johnston’s unpublished data). In addition, our previous flow cytometry results also showed that the *D. japonica* genome size is ~ 1.9 times that of the *S. mediterranea* genome [19]. Thus, the deduced *D. japonica* genome size should be around 1.46 Gb, similar to the genome size estimated from the kmer frequency graph.

### Fosmid sequences revealed some features of the *D. japonica* genome

To display genome features visually, we screened out several individual fosmid clones of certain genes by a colony multiplex qPCR-based 3S3DBC method [43] from the *D. japonica* genomic DNA fosmid library and sequenced them by the Sanger method. We then aligned next-generation sequencing DNA and RNA reads to those fosmid sequences.

**Fig. 2:**
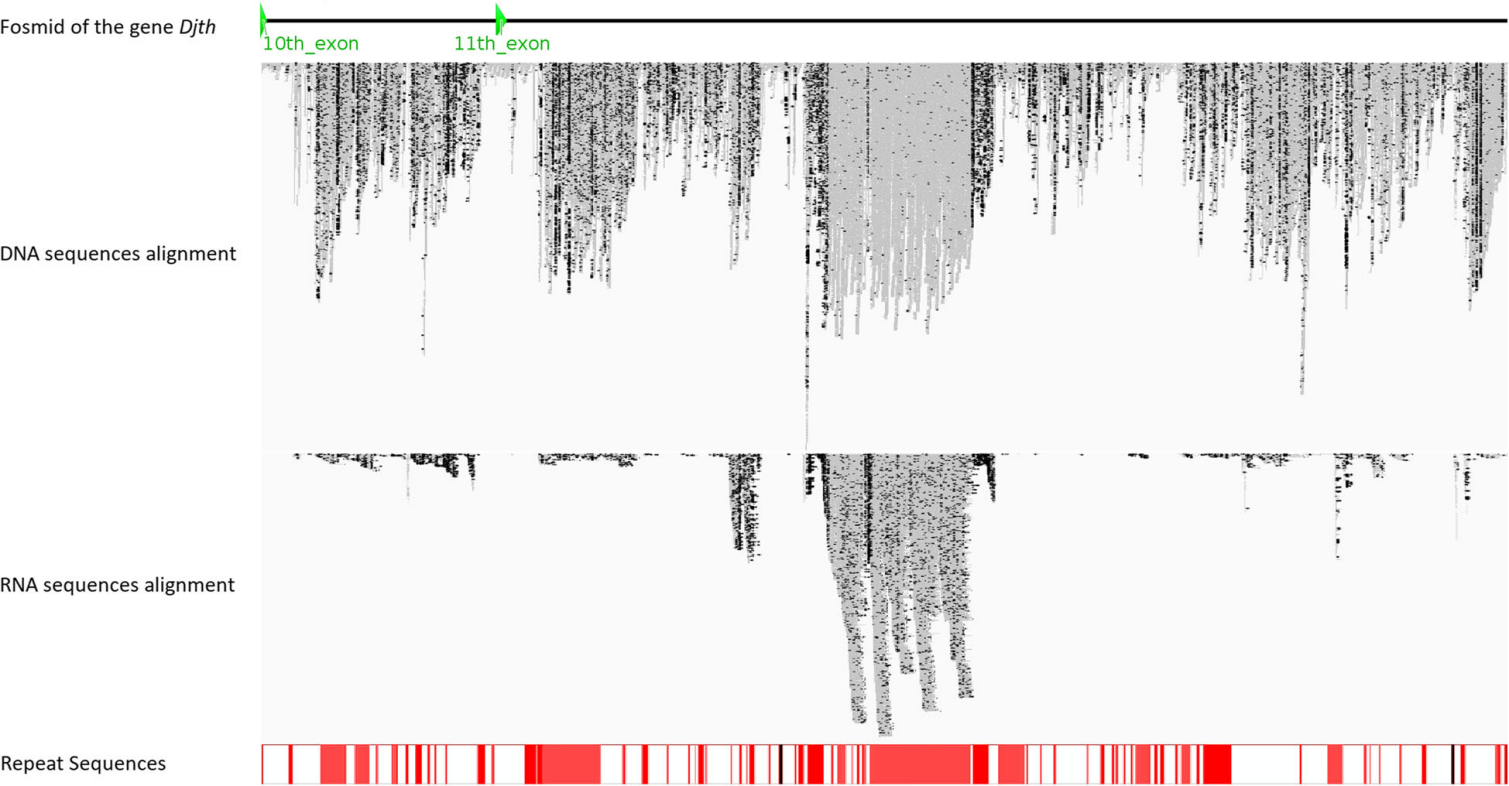
One fosmid insert sequence of the gene *Djth* (DJF-016O13). This figure shows the alignment of genomic DNA and RNA sequencing reads to one fosmid insert sequence of the gene Djth (DJF-016O13) and the repeated elements annotation of this fosmid sequence by Repbase. Green arrowheads show the10th and 11th exon of the Djth gene on the fosmid insert sequence. Grey spots show matches between the sequencing reads and the reference fosmid sequence, and black spots show mismatches. Red bars represent repetitive sequences

In one representative example, the alignment between DNA sequencing reads and one fosmid (named DJF-016O13) insert sequence of the gene *Djth* (Fig. 2) showed that only the sequences around the coding region aligned closely (58 reads mapped on coding regions, 55 of which matched perfectly), but sequences outside of coding regions are highly variable, which is caused by repetitive sequences (41% of the sequence were marked as repeated sequences, as shown by red bars in Fig. 2). Alignment between RNA sequencing reads and the fosmid insert sequence also indicated that a large fraction of the repeated sequences are transcribed, suggesting that these repeats are consist of retrotransposons, which iswas also in accord with the Repbase [44] annotation of this fosmid (Additional file 2). Furthermore, we found several SNPs even in the coding region, as previously reported by Nishimura et al. [19].

### Preliminary genome assembly using SGS short reads

Because most of our genome sequencing data are short pair-end reads obtained from second-generation sequencers, *De Bruijn* graph-based genome methods could be used to assemble the genome. We used Allpath-lg [21] (not completed due to superabundant memory requirement), SOAPdenovo [22] and Velvet [23] to assemble the clean genome sequencing reads after quality control. However, there is a strong possibility that the complexity of the *D. japonica* genome would severely interfere with genome assembly. Not surprisingly, the de novo assembled results were fragmented (scaffold N50 < 1000 bp, Additional file 3) irrespective of the assembler used, parameters set, or the stringency with which the input data were trimmed. Accordingly, the combination of heterogenesis and repeated sequences of the genome disturbed de novo genome assembly using only short SGS data. To address this challenge, a new assembly strategy was required.

### Genome *de novo* assembly using merged pseudo-long reads

**Fig. 3:**
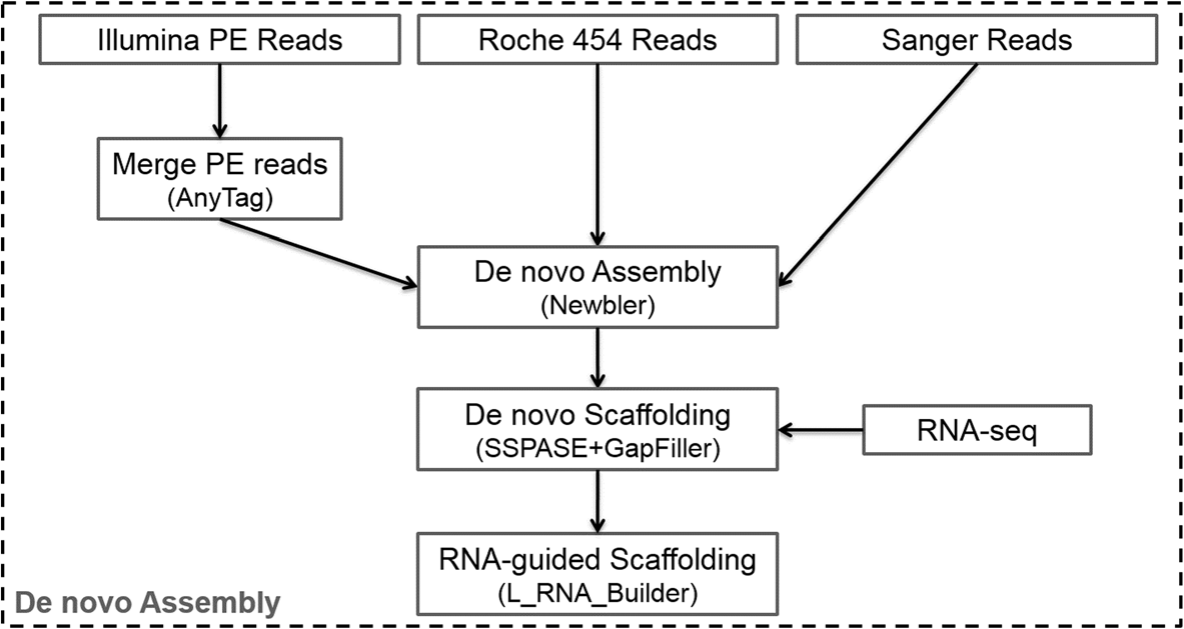
*D. japonica* genome assembly workflow

The result from *de Brujin* graph-based de novo assembly using short sequencing reads was not satisfactory. Thus, we developed a more convenient pseudo-long read strategy (Fig. 3), taking advantage of the numerous pre-existing short sequencing data. In brief, all short pair-end sequencing reads from small insert libraries (~ 180 bp, 200 bp, 250 bp, 450 bp) were, at first, locally assembled into precise pseudo-long reads [24] with average length ~ 452 bp, which is as long as common Roche 454 sequencing reads. Secondly, genome contigs were generated from all Sanger long reads, Roche 454 long reads, and the newly produced pseudo-long reads by using an overlap-layout algorithm. Mate-pair sequencing reads from long-insert libraries (~ 3 kb, ~ 8 kb and 20 kb) were used to link the contigs and generate scaffolds of the *D. japonica* genome [45]. Finally, all pair-end and mate-pair information from the sequencing was utilized for gap closure of the previously assembled scaffolds [46]. An optional super-scaffold generation step could also be performed by further linking between scaffolds [28] using mRNA evidence, which contains the orientation and order information of the exon sequences, which could be exploited to help us determine the orientation and order of previously obtained scaffolds containing those exon sequences. Although the distance information between the RNA-guided scaffolds is ambiguous, these scaffolds are of great value for the subsequent genome annotation.

Finally, by assembling the new pseudo-long reads, we obtained an assembled genome size 1.56 Gb, with a contig N50 size of 1.4 kb, a scaffold N50 size of 23.2 kb, and an RNA-guided super-scaffold N50 size of 27.7 kb (Table 2). We called this assembly result DjGenome ver 1.0. Over 75% of the assembly was covered by scaffolds ≥10 kb. Although genome complexity hampered the assembly, this new strategy yielded a much-improved genome assembly, whose scaffold N50 length was more than 20 times longer than the previous short read-based assembly.

**Table 2:** Summary of the *D. japonica* genome assembly

Terms	Contigs	Scaffolds	RNA-guided Super-scaffolds
N50 (bp)	1408	23,204	27,741
Longest (bp)	186,265	760,010	760,010
Total number (> 1Kb)	286,283	135,705	126,524
Total number (> 10Kb)	466	43,707	38,208
Total number	951,280	213,090	202,925
Total size (Gb)	0.9	1.56	1.56

### Assessment of the *D. japonica* genome

The completeness of the assembled scaffolds was assessed by gVolante [47]. In total, 91.13% of the 248 core conserved eukaryotic genes were covered by the assembled scaffolds, indicating a high level of completeness of the *D. japonica* genome assembly.

**Fig. 4:**
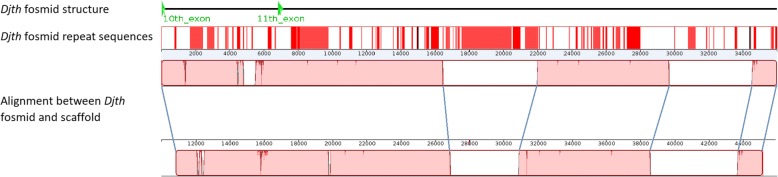
Alignment between the *Djth* fosmid insert sequence and its corresponding genome scaffold. In the illustration of the *Djth* fosmid structure, green arrowheads show the 10th and 11th exons of the *Djth* gene in the fosmid (see Fig. 2). In the illustration of the *Djth* fosmid repeated sequences, red bars represent repetitive sequences. In the illustration of the alignment between *Djth* fosmid and scaffold, pink blocks show matched sequences between the fosmid and the corresponding scaffold, while white blocks show mismatches, which were mainly caused by gaps in the scaffold

We also assessed the genome assembly results by using fosmid and RNA sequencing data. After sequencing using first-generation Sanger technology and quality control, 89,721 fosmid end sequences (FESs) were generated. All of these sequences were mapped back to the assembled reference genome scaffolds using Bowtie [48]; 80.6% could be mapped back to the reference genome. Some complete fosmid insert sequences were also obtained by Sanger sequencing and assembled by CAP3 [49]. After aligning them to the assembled genome scaffolds, a representative example showing the alignment between the *Djth* fosmid sequence (DJF-016O13) and its corresponding genome scaffold matched well, although some mismatches existed and large gaps in the scaffold caused by repeated sequences could not align to the fosmid (Fig. 4).

Moreover, the *D. japonica* transcriptome was obtained by assembling Roche 454 RNA sequencing reads derived from our previous results [19]. The assembled transcriptome was ~ 34.78 Mb in size, and it contained 19,543 genes (with 25,566 transcripts). All the transcripts were aligned to the assembled genome scaffolds using BLAT [32] with identity ≥95%. In the results, ~ 99.4% of all transcripts could find their corresponding genome scaffolds, and ~ 97.8% of all transcripts’ bases were covered by the genome, which also indicated that this assembled genome covered nearly all coding gene regions (Table 3).

**Table 3:** Summary of the *D. japonica* transcriptome assembly and genome scaffolds coverage

Term	Value
Statistics for isotig length	
Min isotig length (bp)	62
Max isotig length (bp)	17,446
N50 isotig length (bp)	1792
Statistics for numbers of isotigs	
Number of isotig groups	19,543
Number of isotigs	25,566
Number of isotigs > = 1 kb	13,256
Genome scaffold coverage of isotig number	~ 99.4%
Statistics for bases in the isotigs	
Number of bases in all isotigs	34,777,653
Number of bases in isotigs > = 1 kb	27,150,697
Genome scaffold coverage of isotig bases	~ 97.8%

Thus, assessment of our *D. japonica* genome assembly by comparison with FESs and the transcriptome showed the relative completeness of the draft genome, especially of the coding region. However, due to the heterogenous and repetitive sequences, some gaps and chimeric scaffolds may still exist.

### Repeated sequences and genome annotation

Repeated sequences were identified by using both RepeatModeler and RepeatMasker (http://www.repeatmasker.org/). RepeatModeler was used to build the consensus models of putative interspersed repeats as a new repeated sequence library based on the genome. RepeatMasker was used to search the planarian genome against the combined library Repbase. The two results were integrated to gain a comprehensive analysis of repeats in the *D. japonica* genome. Approximately 39.7% of the assembled genome sequences were marked as repetitive elements (Table 4). This rate is lower than that estimated by kmer frequency analysis. Ends of contigs were also often occupied by tandem repeats. This suggests that repetitive sequences interrupt genome assembly and make it difficult to assemble a draft genome. Moreover, except for unclassified repeats in the assembled genome, the majority of repeated elements were retrotransposons and DNA transposons, which agreed with our observations from the fosmid survey.

**Table 4:** Summary of repeated elements in *D. japonica* genome

Repeated Elements	Numbers of elements^a^	Length (bp)	Percentage of genome (%)
Retrotransposon	260,765	122,888,499	7.85%
LTR-Retrotransposon	194,395	101,831,050	6.51%
Non-LTR Retrotransposon	66,370	21,057,449	1.35%
DNA Transposon	323,715	109,645,290	7.01%
Unclassified	1,602,282	355,858,117	22.74%
Small RNA	4762	993,638	0.06%
Simple repeats	411,512	27,237,668	1.74%
Low complexity	80,856	4,603,818	0.29%
Total count	2,683,892	621,227,030	39.69%

^a^Most repeats fragmented by insertions or deletions were counted as one element

**Fig. 5:**
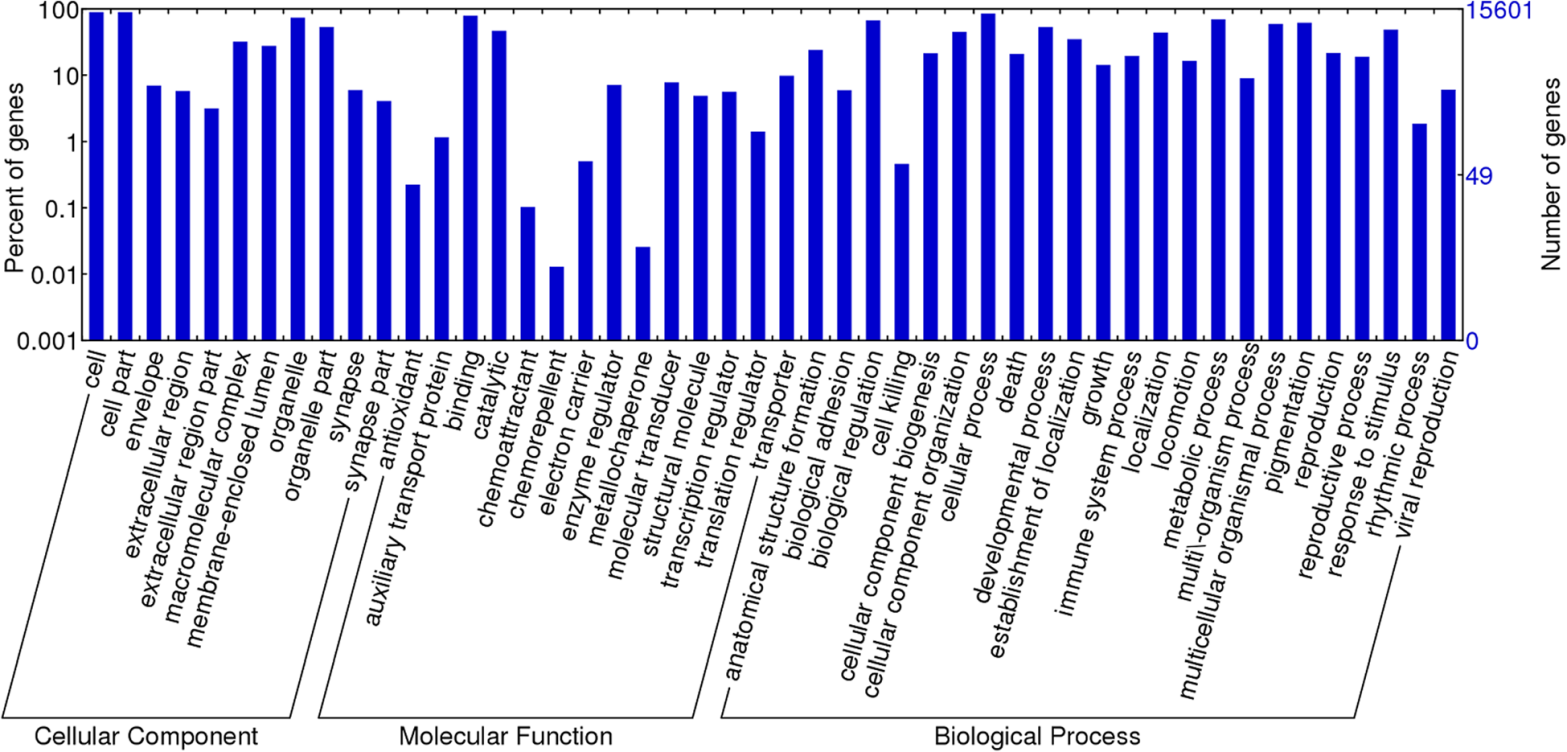
Category of gene ontology annotation

To predict coding genes in the *D. japonica* genome after masking repeated elements, we used evidence-based prediction followed by de novo prediction. In the evidence-based method, 2,857,787 long RNA sequences (including ESTs, 454 sequences and assembled transcripts) were aligned against the *D. japonica* genome with BLAT (identity > 95%) [32]. The best alignment output result of each RNA sequence was taken as evidence of a coding region in the genome, and the information was further used by employing Augustus [50] to help de novo gene prediction. Finally, we identified a total of 23,997 coding genes in the *D. japonica* genome and annotated them by Blast2GO annotation [51]. 15,601 genes were assigned a gene ontology (GO) annotation (Fig. 5 & Additional file 4).

The comprehensive gene and protein prediction were performed by MAKER genome annotation program [29], which employs several algorithms for annotating genomic regions such as repeats and homologous regions to genes and proteins of other organisms. Maker reported 8,958,273 hits within 179,194 (88%) unique scaffolds of our genome assembly. There were homologous regions to 138,931 unique proteins in Swiss-Prot database from 5572 organisms (Table 5).

**Table 5:** The number of hits within the *D. japonica* genome assembly by different programs of MAKER genome annotation platform

Program	Number of hits
Blastn	2,502,462
Blastx	1,108,696
Est2genome	2,368,346
Protein2genome	721,473
Repeat masker	2,255,084
Repeat runner	2212

So far there are only three experimentally confirmed protein sequences from *S. mediterranea* in Swiss-Prot database, all of which had homologous hits in our *D. japonica* reference assembly. We separately run Blastx on the unconfirmed 1366 protein sequences of *S. mediterranea* in Uniprot database and found homologous hits to 1129 (82%) of them. Moreover, from our *D. japonica*’s genome, we found several conserved critical protein coding genes which were recently reported missing from the genome of *S. mediterranea* in the report by Grohme et al. [52], including MAD1L1, BUB1, ANAPC7, NCAPD3, LIG3, NFE2L1, ACADM, et al. Thus, the completeness of our assembled genome and the conserved genes it contains make *D. japonica* a good model animal for further research.

### Database of DjGenome ver 1.0

For easy access to the assembly and annotation data, we prepared a *D. japonica* online genome database (http://www.planarian.jp/). It allows users to download the genome and the transcriptome results, and features a graphical interface for browsing the genome using the JBrowse platform [53, 54]. In addition to the genome assembly, annotated regions by MAKER, Augustus, and Blastx hits to Swiss-Prot are available different tracks.

### Discovery of conserved non-coding elements in planarian

Because of protein functional constraints, coding regions are expected to exhibit sequence conservation between related species. In addition, some non-coding elements also show functional constraints, and such conservation outside of exons can be detected by cross-species comparison. In vertebrates, conserved non-coding elements (CNEs) were found to include transcription factor binding sites [55], and they are accepted as beacons of gene regulatory elements [56]. Planarians have been used as a model animal for regeneration and stem cell research for many years. Although the planarians *D. japonica* and *S. mediterranea* exhibit strong morphological, physiological and functional similarity, their evolutionary positions are distant (even further than the distance between human and chicken [57]) and their gene sequences also have large differences. Genome sequence comparison between these two planarian genera could help us to find common CNEs among planarians, which would light the way to studying molecular regulatory networks in planarian. By combining the results from NUCmer [34] and Blastn procedures (both local Blastn [35] and online Blastn at the website of *S. mediterranea* genome database [36]), we identified CNEs from 33,924 *D. japonica* genome scaffolds that have mRNA evidence matched on 9738 *S. mediterranea* genome scaffolds.

### A CNE is a regulatory element of the *Djndk* gene

In 2002, the planarian gene *Djndk* (a homolog of the vertebrate *fgfrl1* gene), was shown to play a crucial role in brain formation during planarian regeneration [58, 59]. However, although more than 10 years have passed since then, the regulatory elements that restrict the expression of *Djndk* to the brain region are still unknown. To test whether the CNEs we identified by genome comparison are indeed regulatory elements, and with the hope of finding regulators of the *Djndk* gene, we took the gene *Djndk* as an example for CNE functional analysis.

**Fig. 6:**
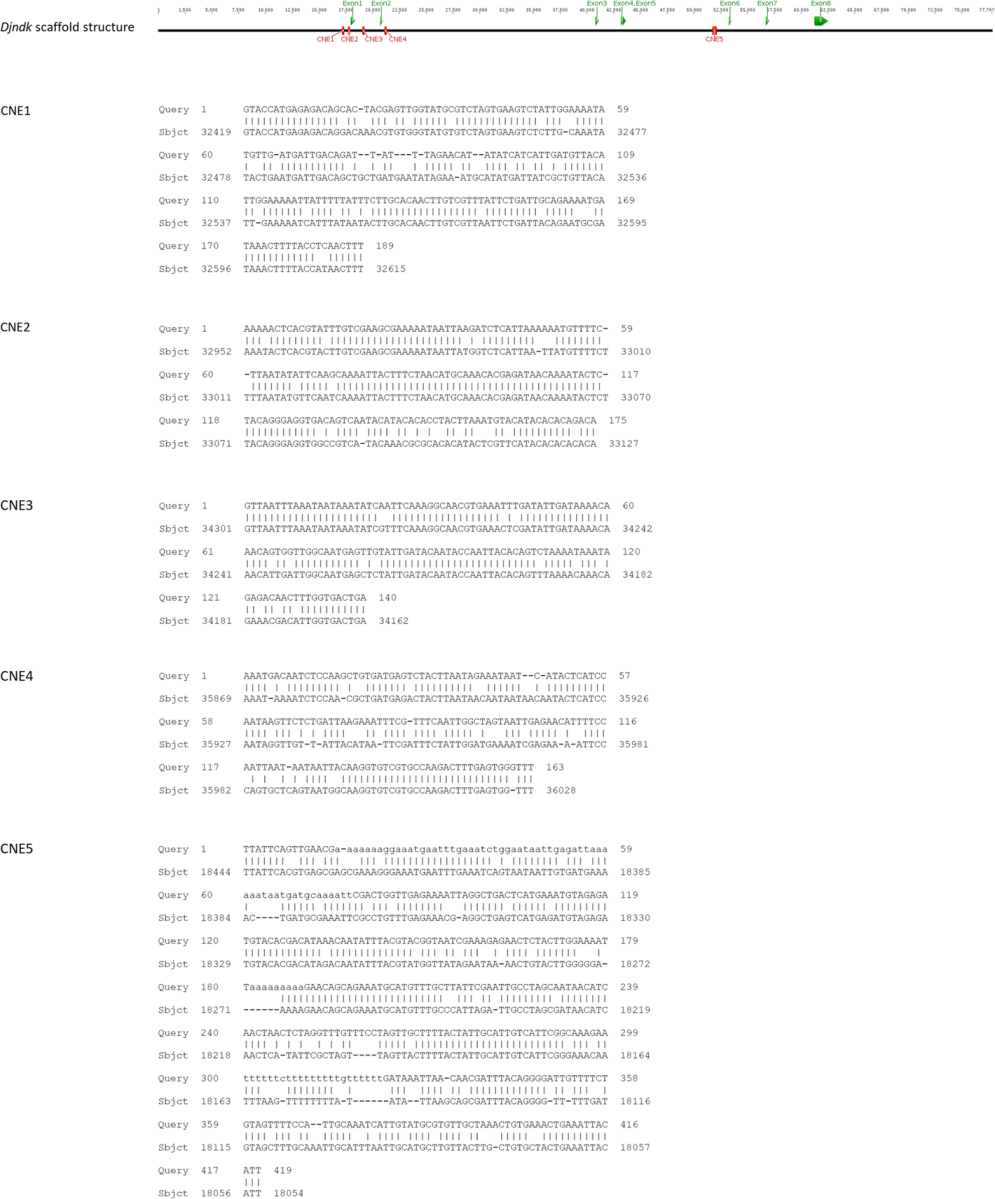
Conserved non-coding elements between *D. japonica* and *S. mediterranea*

**Fig. 7:**
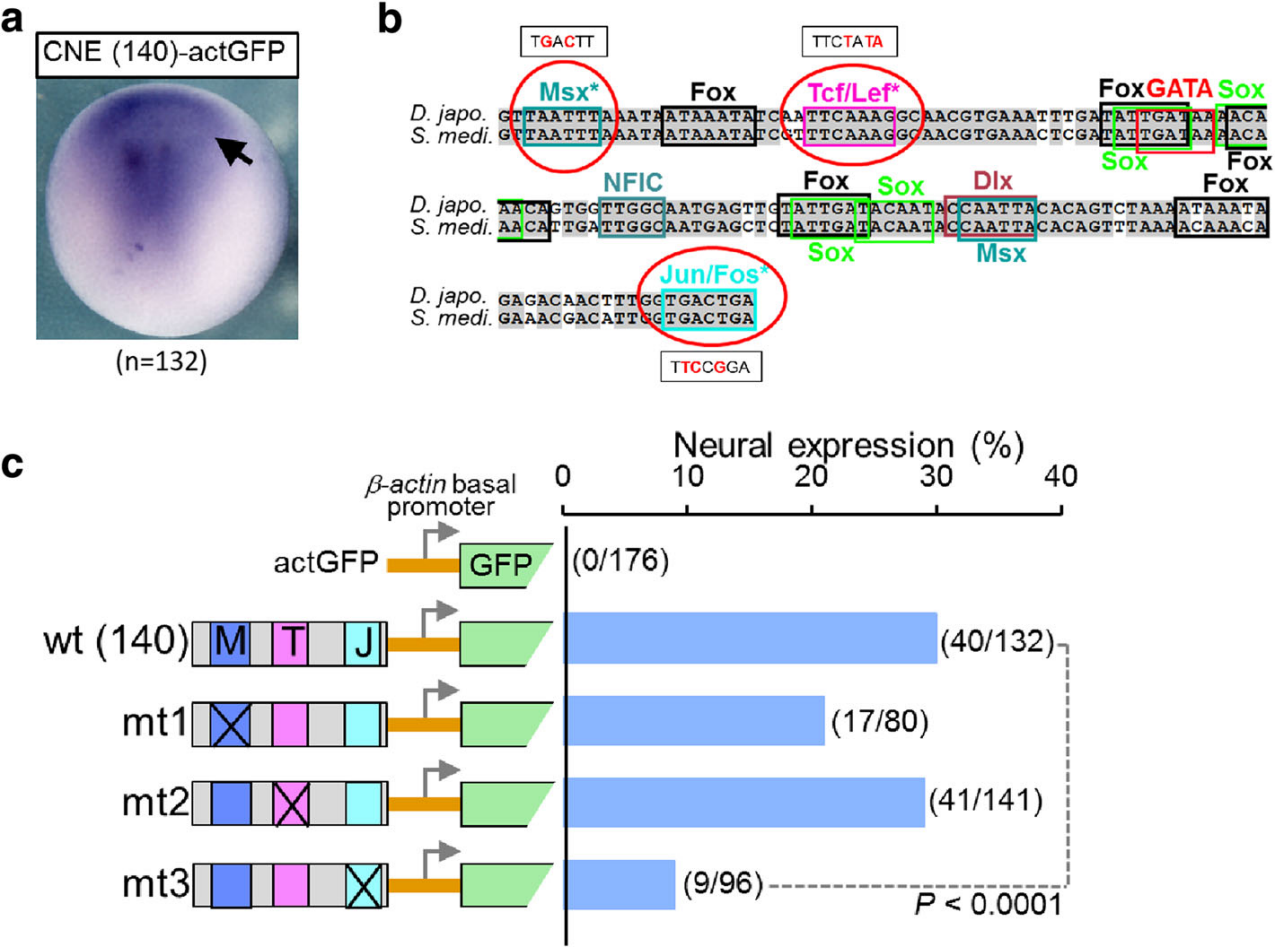
Transgenic experiments suggested that *Djndk* CNE3 might be a regulatory element. **a** The arrowhead shows CNE3 inserted actGFP vector, CNE (140)-actGFP driven GFP to express in the anterior region at the end of gastrulation in transgenic *Xenopus* embryos (*n* = 132). **b** Putative transcription factor-binding motifs are boxed in different colors; those subjected to mutation analysis are indicated by asterisks. The detailed point mutation design of three transcription factor-binding motifs are shown by red colored words. **c** Mutation analysis of CNE3 (the 140 bp element). actGFP is an empty reporter construct that contains the *β-actin* basal promoter; wt (140) is the construct of CNE (140)-actGFP used in (**a**); mt1, m2 and mt3 are point mutations (Msx (M), Tcf/Lef (T), and Jun/Fos (J)) generated from wt (140), and the detailed mutation design is shown in (**b**). The bar chart shows the percentage of the embryos that showed GFP expression in the neural plate among total developed embryos injected with the vector constructs. Actual numbers of GFP-positive cases and total numbers of scored embryos are indicated in parentheses. The chi-square test showed that the percentage of positive cases in the wt (140) and the Jun/Fos mutant constructs are significantly different (*P* < 0.0001), whereas the differences observed in other cases were not significant (*P* > 0.05)

There is only one corresponding scaffold (77.80 kbp) in our assembled genome of *D. japonica,* which contains all 8 exons of the gene *ndk* and its flanking sequences on both 5′ upstream and 3′ downstream. Two scaffolds, v31.003636 (53.55 kbp, contains exons 1–5 of *ndk*) and v31.014514 (21.89 kbp, contains exons 6–exon 8 of *ndk*), were found from the genome of *S. mediterranea* (Smed Sexual v31 in SmedGD [36]). After genome comparison between those full scaffolds, five CNEs were distinguished (Fig. 6). To examine whether CNEs of the *Djndk* gene exhibit regulatory activity, we focused on CNE3 (140 bp) as a representative example for transgenic expression experiments (Fig. 7), since we found the similar conserved non-coding sequences among vertebrate FGFRL-1 (vertebrate *nou-darake* homolog) loci. The 140 bp CNE3 was inserted upstream of the promoter of a *β-actin* promoter-driven GFP expression vector to form a reporter construct called CNE-actGFP. When this expression vector was injected into *Xenopus laevis* embryos, the GFP expression pattern was localized in the neural-plate-forming region of the embryo, which was especially evident in the anterior region at the end of gastrulation (Fig. 7a). Such expression pattern was reproducibly observed in the normally-developed injected embryos (30%, *n* = 40/132), confirming the discovery of this reporter-gene-expression regulation by CNE3.

By aligning the CNE sequences between *D. japonica* and *S. mediterranea*, putative transcription factor-binding motifs were identified from perfectly or almost matched sequences (Fig. 7b). We made three point mutations in the expression vector in putative Msx(M), Tcf/lef1(T) and Jun/Fos(J) transcription factor binding sites, respectively (Fig. 7b). Statistical analysis by the chi-square test showed that the percentage of positive cases driven by the Jun/Fos mutant binding site constructs were significantly reduced compared to the wild-type CNE “wt” (140 bp) (*P* < 0.0001). These results suggest that the 140 bp CNE3 might be a regulatory element of the *Djndk* gene, and that the Jun/Fos-related transcription factor(s) regulates the expression through this region. Accordingly, CNEs are good candidates of regulatory elements in the planarian genome, and further analysis should be done in the future to thoroughly dissect their roles in gene regulatory networks in planarian.

## Discussion

In this study, we assembled the *D. japonica* draft genome. Our kmer frequency graphs, analysis of fosmid sequences, and our further genome analysis after assembly, taken together with previous heterogenesis observations, indicated that *D. japonica* possesses a hyper-varying genome structure. Although, the planarian clonal line we used for genomic DNA extraction was derived from a re-cloned planarian individual, we recently found accumulation of large number of mutations during planarian asexual proliferation, suggesting that planarian has a property to avoid complete genome stability to adapt changing environment [19], which may account for the broad distribution of *D. japonica* across Russia, China, Korea, Japan, and other East Asian countries [2]. One possibility is that, in the kmer frequency graphs, those kmers came from differences between the genome sets of different cells even within one individual. Recently, in planarians we reported that activation of retrotransposons occurs in the course of cell differentiation from the pluripotent stem cell state [60]. In addition, the genomic DNA used for sequencing was pooled from ~ 200 individuals, which would add more complexity in the samples using for the *D. japonica*’s genome project and make the genome assembly much more challenging.

During genome assembly, simply using the SGS short sequencing data and assembling by the *de Bruijn* graph-based method did not produce good assembly results. Alternative solutions for this problem, such as bacterial artificial chromosome (BAC)-to-BAC sequencing and fosmid-pooling sequencing [61] are difficult and expensive. It is well known that long sequencing reads have great advantages over short reads in assembling complicated genomes (long reads can solve the assembly problems caused by repetitive sequences and, to a large extent, relieve the interference caused by heterozygosity) [52], so we executed a new strategy by locally merging short pair-end reads into long reads, and used those long reads to de novo assemble the genome by an overlap-layout-based method. The final assembly result was significantly improved. This strategy could be further used for assembling other complicated genomes that present similar difficulties when only short pair-end reads are available. Besides the strategy of merging short pair-end reads into pseudo-long reads, much longer sequencing reads from third generation sequencing technology (e.g., Pacbio) could facilitate improvement of the genome assembly of *D. japonica* in the future. Moreover, because genomes extracted from different planarian cells even within one individual are possibly heterogenetic, which increased the complexity of the genome structure of *D. japonica* analyzed here and hampered the genome assembly, the usage of single-cell or single chromosome sequencing technology will help reduce the complexity and improve the genome assembly in future research.

CNEs are well accepted as locations of gene expression regulatory elements. In this research, we also proved that one CNE (CNE3) in the *Djndk* gene is a regulatory element that has a binding site for the Jun/Fos-related transcription factors. Previous result showed that Xenopus *Djndk* homologue (FGFRL-1) was expressed in the anterior part of the neural plate of *Xenopus* embryos, which will form the brain during development [62]. However, in the present transgenic expression experiment, CNE3 could only restrain the reporter gene expression to within the whole neural plate, but not to within the anterior part of the neural plate. This suggests that some other regulatory factor(s) are present in addition to CNE3. We found five CNEs, and thus some other CNE(s) could also be regulatory elements for transcription factors, or possibly other regulators such as miRNA may also play a part in regulating *Djndk* gene expression. In addition, CNE3 in planarians has a similar counterpart in the human, mouse, and frog *ndk*-homologous genes, and thus we expect that the regulation of the *ndk* gene and even *ndk*-related aspects of the brain formation mechanism might be conserved between vertebrates and invertebrates.

## Conclusions

We presented the first draft genome of the planarian *D. japonica*. Although the draft genome is still fragmented, it covers nearly all gene coding regions and is useful for helping to identify CNEs in the planarians, which will facilitate research on the gene regulatory networks of planarians.

## Additional files

Additional file 1: Quality control of sequencing reads. (PDF 255 kb)

Additional file 2: Repbase annotation of repeat sequences on the fosmid DJF-016O13. (XLSX 15 kb)

Additional file 3: De novo assembly by De Bruijn Graph algorithm. (XLSX 10 kb)

Additional file 4: Gene ontology annotation. (XLSX 11 kb)
