# Supplementary material for: Draft genome of Dugesia japonica provides insights into conserved regulatory elements of the brain restriction gene nou-darake in planarians
Source: Zoological Lett. 2018 Aug 29;4:24. doi: 10.1186/s40851-018-0102-2 (PMC6114478; doi:10.1186/s40851-018-0102-2)
Supplement: Supplementary file 1 — Quality control of sequencing reads. (PDF 255 kb) [file 40851_2018_102_MOESM1_ESM.pdf]

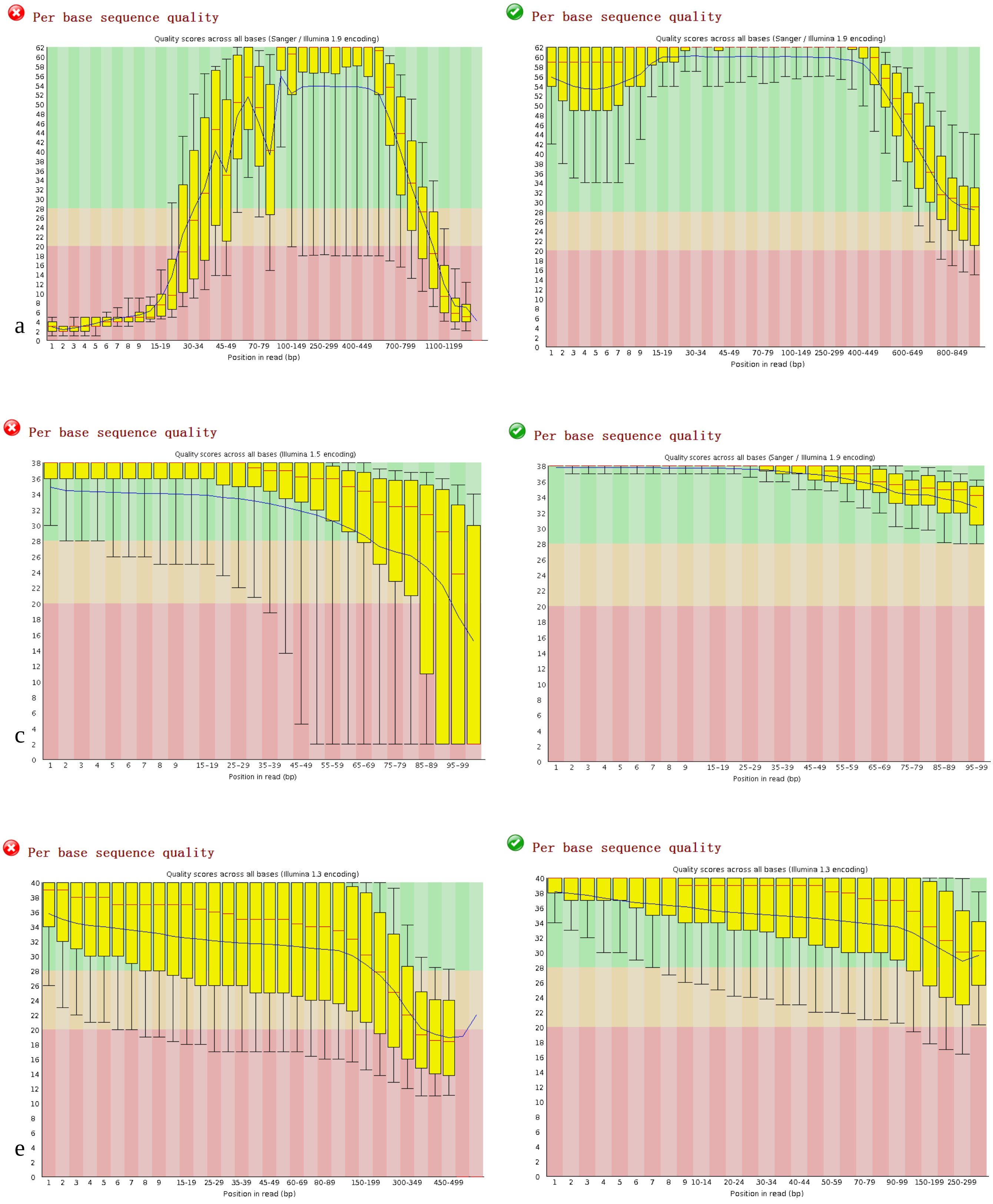

Additional File 1. Quality control of sequencing reads

Sanger (a,b), Illumina (c,d), and Roche 454 (e,f) sequencing data
